# Clinical, patient-reported, radiographic and magnetic resonance imaging findings 11 years after acute posterior cruciate ligament injury treated non-surgically

**DOI:** 10.1186/s12891-023-06480-0

**Published:** 2023-05-09

**Authors:** Jamie S Brown, Krister Mogianos, Frank W Roemer, Anders Isacsson, Jaanika Kumm, Richard Frobell, Ola Olsson, Martin Englund

**Affiliations:** 1 Aleris Specialist Care, Ängelholm Hospital, Landshövdingevägen 7E, Orthopaedics, Ängelholm, 26252 Sweden; 2 Operation and Intensive Care Clinic, Halmstad Hospital, Lasarettsvägen, Halmstad, 30233 Sweden; 3 Department of Radiology, Friedrich-Alexander University Erlangen-Nürnberg (FAU) and Universitätsklinikum Erlangen, Maximiliansplatz 3, 91054 Erlangen, Germany; 4 Quantitative Imaging Center, Department of Radiology, Boston University School of Medicine, FGH Building, 3rd floor, 820 Harrison Ave, Boston, MA 02118 USA; 5 Department of Orthopaedics, Helsingborg Hospital, Charlotte Yhlens gata 10, Helsingborg, 25223 Sweden; 6 Clinical Epidemiology Unit, Department of Clinical Sciences Lund, Lund University, Remissgatan 4, Orthopaedics, Wigerthuset, Lund, 22185 Sweden; 7 Department of Radiology, Department of Radiology, Tartu University, Tartu University Hospital, L.Puusepa 8, Tartu, 50406 Estonia; 8 Lund Osteoarthritis Division- Joint injury research group, University Hospital, Lund, 22184 Sweden

**Keywords:** Posterior cruciate ligament, Non-surgical treatment, Long-term outcome

## Abstract

**Background:**

Long-term consequences of posterior cruciate ligament (PCL) injury such as persistent posterior tibial translation and risk of osteoarthritis development are unclear. Additionally, little data is available describing the natural history of structural morphology of the ruptured PCL. The purpose of the study was to determine the long-term outcome after non-operatively treated PCL injury.

**Methods:**

Over 6-years, all acute knee injuries were documented by subacute MRI (median 8 days [5–15, 25th − 75th percentile] from injury to MRI). Twenty-six patients with acute PCL injury were identified of whom 18 (69%) participated in the long-term follow-up after 11 years. Follow-up included radiographic posterior tibial translation (RPTT) determined using the Puddu axial radiograph. weight-bearing knee radiographs, MRI and KOOS (Knee injury and Osteoarthritis Outcome Score).

**Results:**

On subacute MRI, 11 knees displayed total and 7 partial ruptures. At 11 (SD 1.9) years, the median RPTT was 3.7 mm (1.5–6.3, 25th − 75th percentile). Seven knees displayed radiographic osteoarthritis approximating Kellgren-Lawrence grade ≥ 2. All follow-up MRIs displayed continuity of the PCL. Patients with more severe RPTT (> 3.7 mm), had worse scores in the KOOS subscales for symptoms (mean difference 14.5, 95% CI 7–22), sport/recreation (30, 95% CI 0–65) and quality of life (25, 95% CI 13–57) than those with less severe RPTT (≤ 3.7 mm). This was also the case for the KOOS_4_ (22, 95% CI 9–34).

**Conclusion:**

Acute PCL injuries treated non-surgically display a high degree of PCL continuity on MR images 11 years after injury. However, there is a large variation of posterior tibial translation with higher values being associated with poorer patient-reported outcomes.

**Supplementary Information:**

The online version contains supplementary material available at 10.1186/s12891-023-06480-0.

## Background

Posterior cruciate ligament (PCL) rupture is a rare knee injury with an annual incidence of between 2 and 4 per 100 000 persons [1, 2]. Non-surgical treatment has been a well-accepted alternative to surgical reconstruction for many years, however, long term structural consequences are still unclear [3–12]. These include the continuity of the PCL itself as well as the risk of development of knee osteoarthritis (OA). Further, the relationship between persistent posterior tibial translation and long-term clinical outcomes is controversial. Some investigators have reported an association between greater laxity and worse outcome [8, 13] however, many have failed to demonstrate such an association [3, 10, 14–16]. One reason may be that the majority of studies are based on clinical stability tests rather than objective tibial translation measures derived from knee radiographs [17–19].

PCL injuries, including those with other concomitant ligament injury, have traditionally been treated with initial non-surgical management at Helsingborg hospital. Concomitant grade II and III collateral ligament injuries were, in addition prescribed a brace for 4–6 weeks. This study used a prospectively ascertained observational cohort of patients with acute knee injury with the aim of investigating radiographic posterior tibial translation (RPTT), frequency of radiographic OA, PCL continuity as visualized by magnetic resonance imaging (MRI) as well as patient-reported outcome measures 11 years after acute PCL rupture treated without surgery. An additional aim was to explore the potential relationship between RPTT and the other outcomes at follow-up. The hypotheses were that larger RPTT would be associated with poorer patient-reported outcomes and that the majority of PCLs would show continuity on follow-up MRI.

## Materials and methods

### Subjects

Between January 2002 and February 2008, a total of 1145 patients were consecutively registered after seeking care at either the emergency department or outpatient clinic at Helsingborg hospital due to acute knee trauma with hemarthrosis. If there was clinical suspicion of a fracture, the patient was referred for conventional radiography. Knees with fractures seen on conventional radiographs, apart from osteochondral and ligament avulsion fractures, were not eligible for the present study. All patients had their structural injuries determined by knee MRI at a median of 8 days (5–15, 25th − 75th percentile) after injury [1]. From this cohort a total of 33 patients with potential PCL injury were identified.

**Fig. 1:**
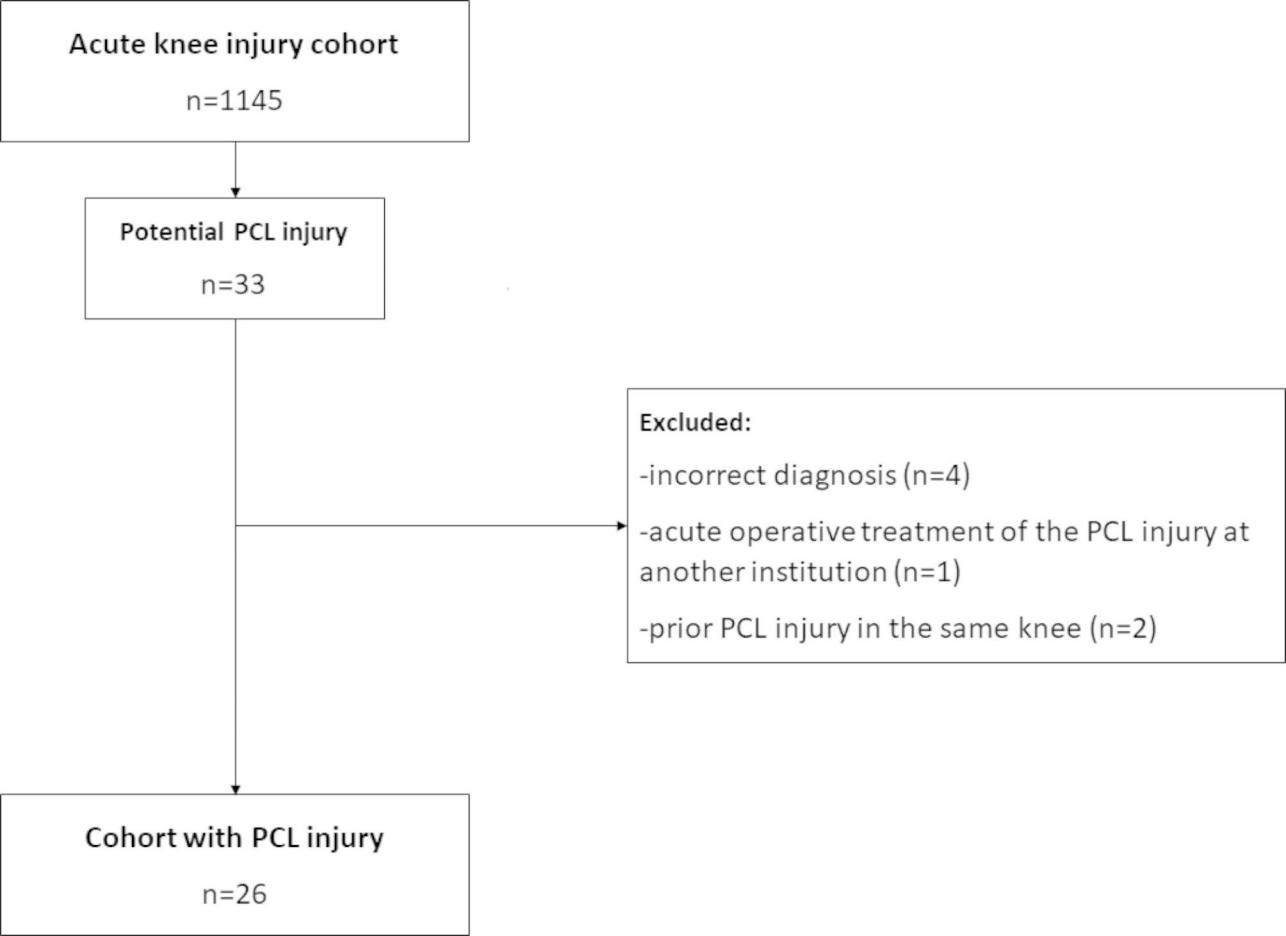
Patients included and excluded

After thorough review of medical records, 7 patients did not meet the inclusion criteria and another 8 were lost-to-follow-up (Fig. 1). Follow-up was conducted between 2017 and 2018.

Medical records were assessed to determine the initial treatment and any registered subsequent injuries to the affected or contralateral knee.

### Baseline knee MR imaging

Patients were examined with 1.0 or 1.5 Tesla MRI [1]. Images were assessed at baseline by musculoskeletal radiologists. MRI findings were classified and collected according to Khanna et al. [20]. PCL injuries were regarded as total ruptures unless a partial rupture was reported.

### Follow-up radiographic examination

Three radiographic projections were obtained of both knees in all 17 patients who attended for examination (one patient completed only patient-reported outcomes). The anteroposterior projections were obtained in a weight-bearing position with the knee flexed 30° – 50° [21]. The Merchant view projection was obtained in standing with the knee flexed 40° – 60° [22]. RPTT was examined using the Puddu axial radiograph using the contralateral knee as the reference [17].

### Radiographic assessment of images

#### Radiographic knee OA

Antero-posterior, lateral and Merchant view radiographs were classified for radiographic OA according to the OARSI-atlas in the tibiofemoral and patellofemoral joint [23] by a musculoskeletal radiologist. In agreement with previous publications the presence of radiographic OA was determined if any of the following criteria were fulfilled, approximating Kellgren-Lawrence grade 2 or worse [24, 25]:

- joint space narrowing (JSN) of grade 2 or more.
- the sum of the two marginal osteophyte grades from the same compartment ≥ 2.
- grade 1 JSN in combination with a grade 1 osteophyte in the same compartment.

#### Radiographic posterior tibial translation (RPTT)

RPTT was quantified as the difference in posterior tibial translation between the affected and unaffected sides in mm on the Puddu axial radiograph [17]. Since two of the 17 patients who attended for radiographic follow-up had suffered a PCL injury to the contralateral knee after their original injury, RPTT measures were obtained from 15 individuals.

A low RPTT corresponds to little sagittal laxity whereas a high RPTT corresponds to large sagittal instability. For comparative analyses, those with RPTT at or below the median value were classified as having low RPTT and those above median as having high RPTT.

#### Continuity of the PCL at follow-up

Images were interpreted by a fellowship trained sports knee and shoulder surgeon (JSB). The PCL was defined as having continuity when low-intensity signals that represented the PCL were shown to be continuous from the femur to the tibia regardless of the shape and configuration [26, 27].

### Patient reported outcome measures

Knee function and symptoms were evaluated using the Knee injury and Osteoarthritis Outcome Score (KOOS). The KOOS_4_ was used to measure the overall patient reported outcome. This is an average score of four of the five KOOS subscales (pain, symptoms, sports and recreation, and quality of life). It excludes the activities of daily living subscale to avoid a ceiling effect given that this group of patients are usually young and active [28, 29]. General health status was evaluated using the EQ-5D VAS. Activity level was assessed with the Tegner activity score and Activity Rating Scale (ARS) [30, 31].

### Clinical examination

At long-term follow-up height and weight were recorded and range of motion and ligamentous stability were assessed. Valgus/varus stability was graded according to Fetto & Marshall [32]. Lachman was graded as per Hefti et al. [33]. Posterior drawer was graded according to Rubenstein et al. [34].

### Statistical analysis

Microsoft excel (2016) and SPSS statistics (version 27) were used to analyse data. Comparisons of patient relevant outcome were evaluated using analysis of variance to produce means with 95% confidence intervals (CI’s). Differences between groups were evaluated using independent samples T-tests and Mann-Whitney U-tests and presented as the differences between means or medians (Lehmann- Hodges estimates) respectively with 95% CI´s. Intra- and inter-rater reliability for tibial translation were assessed using ICC.

## Results

### Cohort followed-up

Eighteen patients (15 men), with a mean age of 33 (SD 14.2) years at injury were followed-up at a mean of 11 (SD 1.9) years after acute PCL rupture (Table 1). The majority were injured during sporting activities (n = 13). Seven patients sustained partial and 11 total PCL ruptures. Nine patients had also suffered injury to other ligaments at the time of injury (Table 2). The characteristics of the cohort followed-up (n = 18) was similar to the cohort as a whole (n = 26) (Table 2).

**Table 1:** Findings at follow-up

Findings at follow up	n = 18
Age at follow-up, mean years (SD)	45 (13.8)
Time from injury to follow up, mean years (SD)	11 (1.9)
Total PCL rupture at baseline	11
Partial PCL rupture at baseline	7
BMI, mean (SD)	26.2 (4.0)
Range of motion, mean degrees (SD)	137 (5.9)
Clinical signs of joint effusion, n	0/17*
Positive posterior drawer test (grade 1–3), n	12/17*
KOOS mean (SD)	
Symptoms	86 (11)
Sport	76 (26)
Pain	89 (15)
ADL	93 (9)
QOL	74 (24)
KOOS_4_	81 (17)
Tegner median (25th -75th percentile)	4 (2.75-7)
ARS median (25th -75th percentile)	6 (0–12)
RPTT median (25th -75th percentile)	3.7 (1.5–6.3)
PCL continuity on MR images	14/14^†^

*Missing data for n = 1 patient. ^†^Four patients did not undergo MRI

**Table 2:** Characteristics at baseline. * Grade 2 or 3 as visualized on knee MRI

	Cohort with PCL injury n = 26	Patients followed up n = 18
Age at injury, mean years (SD)	30 (12)	33 (14.2)
Males, n (%)	21 (81)	15 (83)
Sports injury, n (%)	19 (73)	13 (72)
Concomitant ligament injury, n		
ACL (total)	10	4
ACL + MCL*	*5*	*2*
ACL + MCL*+LCL*	*1*	*1*
MCL*	4	4
LCL*	1	1

### Treatment and subsequent injuries

None of the patients followed-up were treated operatively for either PCL injury or collateral ligament injury however, 2 patients underwent a subsequent ACL reconstruction and 4 patients were treated with partial meniscal resection. Three of these 4 cases were related to meniscal injuries identified at the time of the subacute MRI.

None of the patients in the study were treated with a dedicated PCL brace however, patients with associated grade 2–3 collateral ligament injuries (n = 8) were treated in a hinged knee brace (0–90 degrees) for 4–6 weeks.

Two patients sustained a contralateral PCL rupture over the follow-up period. No other subsequent knee injuries to either knee were recorded in the cohort.

### Outcomes in relation to RPTT and degree of ligament injury

Inter-rater reliability was determined by two assessors measuring RPTT for 14 patients. Intra-rater reliability was determined using re-assessment of radiographs from 14 patients 49 days apart. Intra class correlations (ICC) were excellent with 0.96 (95% CI 0.87–0.99) and 0.99 (95% CI 0.98-1.00) for inter- and intra-rater reliability respectively.

**Fig. 2:**
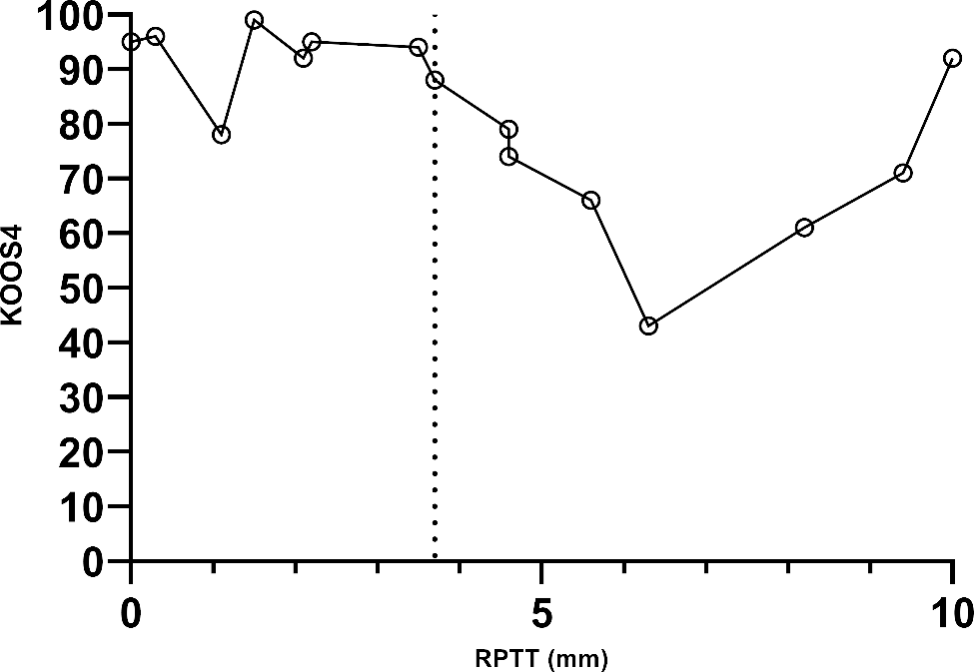
KOOS_4_ score vs. RPTT. Dotted vertical line represents median RPTT

**Fig. 3:**
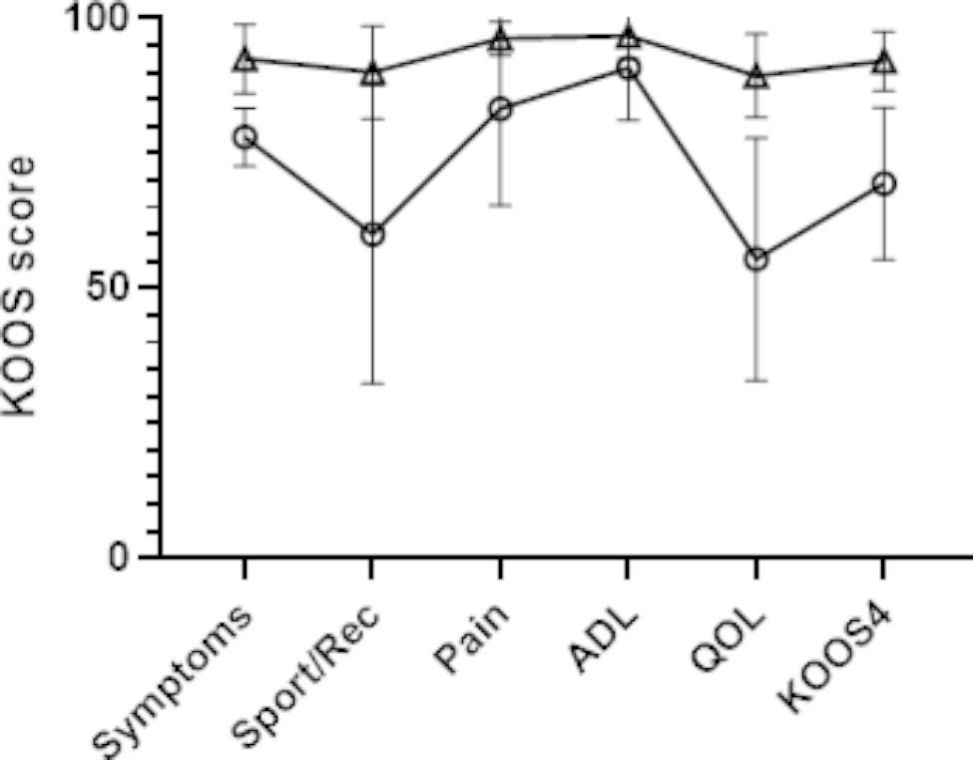
Mean KOOS scores with 95% confidence interval. △ = Low RPTT, ○ = High RPTT, Rec = recreation

Median RPTT was 3.7 (25th-75th percentile 1.5–6.3) mm for the entire cohort (Table 1) with overall decreasing KOOS scores for those with higher RPTT (Fig. 2). Those with high RPTT (above median, n = 7) had lower KOOS scores in general, and results were statistically significantly worse for symptoms (mean difference 14.5, 95% CI 7–22), sport/recreation (median difference 30, 95% CI 0–65) and knee related quality of life (median difference 25, 95% CI 13–57) than those with low RPTT (at or below median, n = 8, Fig. 3). This was also the case for the KOOS_4_ (median difference 22, 95% CI 9–34). There were no corresponding statistically significant differences in the KOOS subscales for pain, or ADL, Eq. 5D VAS or for activity level as measured by the Tegner score and the Activity Rating Scale (data provided in additional data file).

**Fig. 4:**
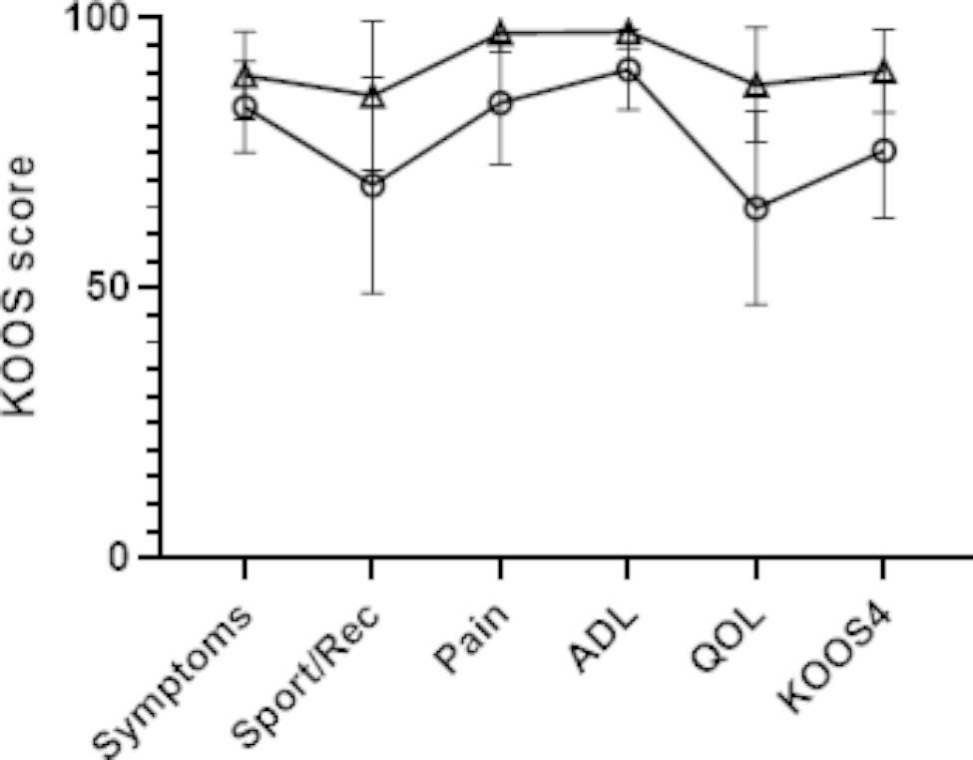
Mean KOOS scores with 95% confidence interval. △ = Partial rupture, ○ = Total rupture, Rec = recreation

Patients who had sustained a partial PCL rupture had a median RPTT of 2.1 (25th-75th percentile 1.1–3.5) mm whereas those who had sustained a total rupture had a median RPTT of 6.0 (25th-75th percentile 3.9–9.1) mm. The KOOS scores were generally lower in patients with total rupture but the differences were rather small and not statistically significantly different (Fig. 4). The other outcomes, Eq. 5D VAS, Tegner score or Activity Rating Scale showed a similar picture between the total rupture and partial rupture subgroups (data provided in additional data file).

There were no statistically significant differences in any of the outcome scores when comparing patients with isolated and combined ligament injuries (data provided in additional data file).

### Radiographic OA

Seven patients had radiographic OA in one or more compartments of the injured knee. Five of these were in the high RPTT group. Three patients also had OA in the uninjured contralateral knee. Four patients showed signs of isolated patellofemoral OA, two of combined tibiofemoral and patellofemoral OA and one of isolated tibiofemoral OA.

### Magnetic resonance imaging

Fourteen of 18 patients underwent an MRI of the index knee at follow-up. All 14 showed continuity of the PCL.

## Discussion

The study suggests that larger RPTT at follow-up is associated with poorer patient-reported outcome. Seven patients displayed radiographic OA with patellofemoral OA being more prevalent than tibiofemoral. Interestingly all 14 knees with acute PCL rupture who underwent MRI follow-up displayed continuity of the PCL 11 years after the injury.

Despite the generally held belief that persistent posterior translation leads to a poorer outcome after PCL rupture this is one of very few studies confirming this notion at long-term follow-up. This may be due to the fact that many previous studies have based their estimation of tibial translation on clinical examination findings rather than objective radiographic measurement [3, 10, 14–16]. There are several methods of measuring RPTT in the literature. The Puddu method was chosen as this is reported to be reliable, less painful than stress radiography and can be carried out relatively quickly with standard radiology department equipment [35]. Studies have shown that PCL deficiency and the resultant instability leads to increased contact pressures particularly in the medial and patellofemoral compartments [36, 37]. This may explain to some degree the symptomatic and radiographic changes demonstrated in this study.

The ability of the PCL to heal has been documented previously [6, 38, 39] and this study adds further weight to this evidence.

The management of PCL injury remains controversial but in recent years brace treatment of acute injuries has gained increasing acceptance [6, 7, 38]. PCL specific braces aim to correct the posterior tibial translation allowing the PCL to heal with the knee in an appropriate position. Given that posterior tibial translation is associated with worse patient related outcome it would seem logical that a treatment that can minimise this phenomenon may improve results. It remains to be proven whether the best method of achieving this is through non-surgical brace treatment or reconstruction.

### Limitations

Despite the study being based on a large cohort of 1145 knee-injured patients collected over a 6-year period there were only 26 patients with acute PCL ruptures and many of these had concomitant ligament injury. As non-operative treatment is utilised for all PCL ruptures, we were not able to compare outcome with a control group of surgically treated patients. The final cohort of 18 patients who attended follow-up included both those with partial and total PCL ruptures. The small sample only makes it possible to ascertain large differences between subgroups. Still, this is mostly a descriptive paper in character. While all the differences presented as statistically significant had p values < 0.05 the research group chose to present comparisons between groups using differences in means/medians and 95% CI´s in order to demonstrate the degree of uncertainty associated with the findings. PCL injury is rare and the study has for the first time performed a systematic follow up of a prospective cohort, including both patient-reported outcomes as well as imaging, 11 years after the injury.

## Conclusions

Acute PCL injuries treated non-surgically, display PCL continuity on MR images 11 years after injury at a very high frequency. However, non-surgical treatment results in a large variation of posterior tibial translation with increased instability being associated with poorer self-reported outcomes.

## Electronic supplementary material

Below is the link to the electronic supplementary material.

Supplementary Material 1

## Data Availability

The datasets analysed during the current study are not publicly available due to ongoing research but are available from the corresponding author on reasonable request.

## Abbreviations

PCL: posterior cruciate ligament
OA: osteoarthritis
RPTT: radiographic posterior tibial translation
MRI: magnetic resonance imaging
ACL: anterior cruciate ligament
MCL: medial collateral ligament
LCL: lateral collateral ligament
JSN: joint space narrowing
ICC: Intra class correlation
KOOS: Knee injury and Osteoarthritis Outcome Score
ARS: Activity Rating Scale
CI: Confidence interval
