# Supplementary material for: Clinical, patient-reported, radiographic and magnetic resonance imaging findings 11 years after acute posterior cruciate ligament injury treated non-surgically
Source: BMC Musculoskelet Disord. 2023 May 9;24:365. doi: 10.1186/s12891-023-06480-0 (PMC10169308; doi:10.1186/s12891-023-06480-0)
Supplement: Supplementary file 1 — Supplementary Material 1 [file 12891_2023_6480_MOESM1_ESM.docx]

Additional data

Statistically insignificant data not shown in results section of manuscript:

*Low vs high RPTT*

KOOS pain: median difference 3, 95% CI -3-33. ADL: median difference 2.5, 95% CI 0-17.

EQ5D VAS: mean difference 1.8, 95% CI -8.4-12

Tegner: mean difference 0.7, 95% CI -2.2-3.7

ARS: median difference 6, 95% CI 0-12

*Partial vs complete rupture*

KOOS Symptoms: mean difference 5.8, 95% CI -5.8-17-3. Sport/rec: median difference 10, 95% CI -10-45. Pain median difference 6, 95% CI 0-33, ADL: median difference 3, 95% CI -1-19. QOL: median difference 19, 95% CI 0-50. KOOS_4_: median difference 12, 95% CI -1-33

EQ5D VAS: mean difference -4, 95% CI -12-4.4.

Tegner: mean difference -0,4, 95% CI -3-2.1.

ARS: median difference 4, 95% CI -2-10.

*Isolated vs combined ligament injury*

KOOS Symptoms: mean difference -6.7, 95% CI -17.8-4.4. Sport/rec: median difference -15, 95% CI -30-10. Pain: median difference -5, 95% CI -30-3. ADL: Median difference -4, 95% CI -13-0. QOL: median difference -12, 95% CI -31-13, KOOS_4_: median difference -8, 95% CI -21-8.

EQ5D VAS: mean difference -3.8, 95% CI -11.9-4.4.

Tegner: mean difference 1.6, 95% CI .0.8-3.9.

ARS: median difference -2, 95% CI -8-4.
